# The Assessment of the Innovativeness of a New Medicine in Italy

**DOI:** 10.3389/fmed.2021.793640

**Published:** 2021-12-08

**Authors:** Filomena Fortinguerra, Serena Perna, Roberto Marini, Alessandra Dell'Utri, Maurizio Trapanese, Francesco Trotta, Scientific & Technical Committee (Commissione Tecnico-Scientifica, CTS) of Italian Medicines Agency-AIFA

**Affiliations:** Italian Medicines Agency, Rome, Italy

**Keywords:** innovativeness, drug therapy, therapeutic need, added therapeutic value, grade

## Abstract

**Objectives:** Starting from April 2017, the Italian Medicine Agency (AIFA) has approved new criteria for defining any new medicinal product with an innovative indication. The purpose of the study is to analyze the activity of innovativeness evaluation according to the new approach, to estimate the weight of each criterion considered for innovativeness definition, and to evaluate how the new approach works in terms of consistency and reproducibility.

**Methods:** A retrospective analysis was performed on the final reports evaluating the drug innovativeness assessment published on the AIFA's website between April 2017 and January 2021. Descriptive statistics, chi-square test, whether the conditions were respected, or Fisher's exact test was used to explore the association between characteristics of drugs and the innovativeness status and the association between the three criteria. Profiles of the decision process and their relationship with innovativeness response were described. In order to evaluate the weight of each criterion in predicting the innovativeness status, a Classification Tree (CT) algorithm was applied.

**Results:** Overall, of the 109 published drugs reports, 37 (33.9%) were recognized as fully innovative, 29 (26.6%) were considered conditionally innovative, while for 43 (39.4%) reports innovativeness was not recognized. Considering the three criteria of the decision process, the added therapeutic value was the only criterion statistically associated with a drug's degree of innovation (*p* < 0.001). The therapeutic need and the quality of clinical evidence were statistically associated (*p* = 0.008) even if only a mild association was observed. The added therapeutic value was the most important variable in predicting the innovativeness status according to the classification tree (CT) model applied, achieving an accuracy of 89.4%. No difference was found between orphans and non-orphan drugs or oncological and non-oncological drugs.

**Discussion:** The added therapeutic value is the most important criterion of the multidimensional approach for the innovativeness status definition of a new medical product. A mild association was found between the therapeutic need and the quality of evidence. Overall, similar decision profiles bring the same evaluation of innovativeness status, indicating a good consistency and reproducibility between decisions.

## Introduction

Currently, the assessment of the innovativeness of a new medicine and the transparent disclosure of the information on the decision-making process are a challenge for many regulatory agencies and health organizations worldwide (1, 2).

In April 2017, new criteria to define the innovativeness of a medicine were approved by the Italian Medicine Agency (AIFA). According to the new approach, as described in our previous paper (3), the decision process used to define the innovativeness of a drug takes into account three criteria, the therapeutic need, the added therapeutic value, and the quality of clinical evidence, which is assessed based on the GRADE (Grading of Recommendations Assessment, Development, and Evaluation) methodology (Box 1).

**Box 1:** The AIFA criteria for assessing a drug's degree of innovation.

**Criterion**	**Level**				
Therapeutic need	Maximum (no alternative therapeutic options available)	Important (alternative therapeutic options available, with no impact on clinically relevant outcomes)	Moderate (alternative therapeutic options available with limited impact on clinically relevant outcomes, and/or uncertain or not satisfactory safety profile)	Poor (alternative therapeutic options available with high impact on clinically relevant outcomes and a satisfactory safety profile)	Absent (alternative therapeutic options available, which are able to slow down the progression of the disease and have a satisfactory safety profile)
Added therapeutic value	Maximum (greater efficacy than alternative therapeutic options (if available) in clinically relevant outcomes, ideally curing the disease or altering its natural history)	Important (greater efficacy based on clinically relevant outcomes, or alternatively one of the following options: i) the drug can reduce the risk of seriously debilitating or life-threatening complications, ii) the drug has a better risk/benefit ratio compared to the alternative therapeutic options, iii) the drug can avoid the use of high risk clinical procedures, iv) the drug can significantly change the natural history of the disease in a subpopulation of patients, v) the drug can provide a clinically relevant added value e.g., in terms of quality of life and disease-free interval, compared to the available therapeutic options)	Moderate (a slightly better efficacy profile or improved efficacy in some patient subpopulations or based on surrogate endpoints and has limited impact on the quality of life. For situations when the lack of a study comparator is acceptable, evidence showing relative efficacy compared to the available therapeutic options should be taken into account)	Poor (greater efficacy only for non-clinically relevant outcomes or based on a poor magnitude of effect. The drug offers minor benefits (e.g., favorable routes of administration) compared to the available therapeutic options)	Absent (no added therapeutic benefit compared to the alternative available therapeutic options)
Quality of clinical evidence^*^	High		Moderate	Low	Very low
Innovativeness status	Fully (innovative)		Conditional (conditionally innovative)		Absent (non-innovative)
Commercial implication	•Funded via “innovative drug fund” •No payback mechanism •Immediate inclusion into regional drug formularies •Benefit duration period up to 36 months		Immediate inclusion into regional drug formularies		No benefits

* *An orphan drug can still be considered innovative, even if the quality of clinical evidence is low or very low when the other two criteria are evaluated as maximum or important. Adapted from (3).*

The final judgment expressed in relation to an individual therapeutic indication of a medicine is formulated on a combination profile deriving from the set of evaluation levels for each criterion. The overall assessment process resulted in a new medicinal product being awarded one of the following three innovative statuses by a specific therapeutic indication: “fully innovative,” “conditionally innovative,” or “non-innovative.” The assessment process was performed by the Scientific and Technical Committee (*Commissione Tecnico-Scientifica*, CTS) of AIFA, which adopted the final decision when deciding on reimbursement and price of a new medicine (or new therapeutic indication); for each assessment, a full report explaining the rationale for the final decision is made publicly available on the Agency's website (4).

The AIFA's model, which is structured and flexible at the same time, ensures that the panel members of CTS consider all the important factors for making a decision, helping them to make discussions about the best available evidence about each criterion and to identify reasons for disagreements. The systematic, rigorous, and transparent assessment process, incorporating explicit decision-making criteria, as in the AIFA's model, aims to address the most common limitations of the decision-making process in healthcare, such as lack of consistency and transparency (5). Furthermore, the choice of AIFA to wield the GRADE methodology to evaluate the quality of clinical evidence within a process of drug innovativeness assessments could support the early identification of the discrepancy between the available clinical evidence at the time of market approval and the need of patients for rapid access to such innovative therapies.

The aim of the present study is to analyze the assessment reports by AIFA's CTS according to the new approach in order to estimate the weight of each criterion considered for the definition of drug innovativeness, the potential interdependence between the three criteria, the consistency between the final decisions emerging from the decision-making process, and their capacity in predicting the final decision in order to understand how the framework works.

## Methods

### Data and Variables of Interest

The final reports evaluating the innovativeness assessment by a specific therapeutic indication of a medicine published on the AIFA's website between April 2017 and January 2021 were collected and analyzed. From each report, the following data were extracted and tabulated: the drug name, the therapeutic indication, the type of medicine (e.g., oncological or orphan drug), the rank assigned to each criterion (therapeutic need, added therapeutic value, quality of clinical evidence), and the final decision on drug innovativeness (“fully innovative,” “conditionally Innovative,” or “non-innovative”).

### Statistical Analysis

Categorical data were summarized as numbers (*n*) and frequencies (%). Profiles of the decision process and its relationship with the decision on drug innovativeness were described in order to investigate the impact of the three domains on the final decision.

A comparison between orphan and non-orphan drugs and oncological and non-oncological drugs was also performed in order to evaluate if the orphan status or an oncological indication have an impact on the weight of criteria in predicting the drug innovativeness.

The association between categorical variables was assessed by Chi-square test, when the conditions were respected, or Fisher's exact test. Moreover, Cramér's V was calculated to assess the strength of the association between the three criteria utilized to define the innovativeness status of a drug. In order to evaluate the weight of each criterion in predicting the innovativeness status, a Classification Tree (CT) algorithm was applied. A CT is a non-parametric model using a tree structure to explore the relationship of a set of potential exploratory variables by developing decision rules for the prediction of a categorical response variable, such as the innovative status. A classification tree consists of three types of nodes: (1)root node, the top node of the tree comprising all the data; (2) splitting node, a node that assigns data to a subgroup; and (3) terminal node, final decision (outcome) (6, 7).

The tree-fitting process initially proceeds by finding the covariate that “best” divides the statistical units into two groups. The “best” split is defined as the one that results in the most homogeneous subgroups with respect to the response variable, according to specific standard measures of goodness-of-fit. The process then separates the observations into the two resulting groups and repeats the splitting process in each of the two groups, a process referred to as recursive partitioning (8). In particular, in the present analysis, the CT was performed using the recursive partitioning classification model which identifies the optimal tree using one procedure divided into two phases: a first phase sees the growth of the trees—according to the maximum decrease in impurity (in our case the Gini index); later the tree in question is pruned through cross-validation method, i.e., minimizing the cross-validated misclassification error across competing sub-trees (the complexity parameter (CP) used to prune the tree was fixed at 0.01) (9–13). The confusion matrix and correct classification rate were calculated to assess the model prediction performance. All analyses were performed using R version 3.5.2 (14). In particular, “rpart” package was used to implement the CT algorithm (15). A two-sided *p*-value less than 0.05 was considered statistically significant.

## Results

Overall, of the 109 published drugs reports, 37 (33.9%) were recognized as fully innovative, 29 (26.6%) were considered conditionally innovative while for 43 (39.4%) reports innovativeness was not recognized (Table 1). Overall, 67 (61.5%) reports concerned oncological indications (24 innovative, 20 conditionally innovative, 23 non-innovative), 41 (37.6%) reports concerned orphan indications (16 innovative, 11 conditionally innovative, 14 non-innovative); and 24 (22.0%) reports were on both oncological and orphan indications (10 innovative, 6 conditionally innovative, 8 non-innovative). While the majority of oncological or orphan drugs were recognized as fully or conditionally innovative, the majority of non-oncological and non-orphan indications were evaluated as non-innovative (32.6%).

**TABLE 1:** Characteristics of drugs criteria considering the drug's degree of innovation.

	**Fully innovative**		**Conditionally innovative**		**Non-innovative^†^**		***p*** -value^*^
	***n*** **= 37**		***n*** **= 29**		***n*** **= 43**		
**Oncological drug**	24	64.9	20	69.0	23	53.5	0.363
**Orphan drug**	16	43.2	11	37.9	14	32.6	0.616
**Oncological and orphan drug**	10	27.0	6	20.7	8	18.6	0.645
**Non-oncological and non-orphan drug**	7	18.9	4	13.8	14	32.6	0.155
**Therapeutic need**							
Maximum	5	13.5	4	13.8	4	9.3	0.081
Important	17	45.9	7	24.1	12	27.9	
Moderate	15	40.5	18	62.1	22	51.2	
Poor	0	0.0	0	0.0	5	11.6	
Absent	0	0.0	0	0.0	0	0.0	
**Added therapeutic value**							
Maximum	1	2.7	0	0.0	0	0.0	<0.001
Important	31	83.8	0	0.0	1	2.6	
Moderate	5	13.5	29	100.0	5	13.2	
Poor	0	0.0	0	0.0	29	76.3	
Absent	0	0.0	0	0.0	3	7.9	
**Quality of clinical evidence**							
High	10	27.0	3	10.3	5	11.6	0.451
Moderate	19	51.4	18	62.1	24	55.8	
Low	7	18.9	6	20.7	9	20.9	
Very low	1	2.7	2	6.9	5	11.6	

*Data were summarized as numbers (n) and frequencies (%).*

* *Chi-square test, when the conditions were respected, or Fisher's exact test was applied to evaluate the association between categorical variables.*

† *For five observations the added therapeutic value was “Untestable” and therefore classified as NA*.

Considering the three criteria of the decision process, the **therapeutic need** was not statistically associated with innovativeness status; however, fully innovative reports presented an important therapeutic need (45.9%) more frequently than the other two groups, conditionally innovative (24.1%) and non-innovative (27.9%). Instead, conditionally innovative assessments reported a “moderate” therapeutic need (62.1%) more frequently than non-innovative (51.2%) and fully innovative (40.5%) reports. Moreover, a “poor” therapeutic value was observed only for non-innovative reports (11.6%). The **added therapeutic value** was the only criteria statistically associated with the drug innovation degree (*p* < 0.001). In particular, among the final reports analyzed, an “important” added therapeutic value was more frequently observed (83.8%) in the indications where full innovativeness was recognized; the added therapeutic value was defined as “moderate” for the totality (100%) of conditionally innovative indications, while a higher percentage of “poor” (76.3%) level of added therapeutic value was observed in the groups of indications defined as non-innovative.

In addition, no difference was found comparing orphan and non-orphan drugs in the weight of each criterion in the assessment process (Supplementary Table 1) and oncological and non-oncological drugs (Supplementary Table 4). For both the drug groups the added therapeutic value remained the only criteria statistically associated with the drug innovation degree (*p* < 0.001).

The **quality of clinical evidence** was defined as “moderate” in most cases, independently from the drug innovation degree. However, fully innovative indications presented more frequently “high” quality level (27.0%) in comparison to conditionally innovative (10.3%) and non-innovative (11.6%) ones, and by contrast, non-innovative drugs presented more frequently “very low” quality level of clinical evidence (11.6%) in comparison to fully and conditionally innovative drugs (2.7 and 6.9%, respectively). As shown in Table 2, the therapeutic need and the quality of clinical evidence were statistically associated (*p* = 0.008), even if only a mild association was observed (Cramér's V: 0.25). Comparing orphans to non-orphan drugs and oncological to non-oncological medicines the association between the therapeutic need and the quality of clinical evidence was not significant (Supplementary Tables 2, 5).

**TABLE 2:** Relationship between criteria utilized in the multidimensional approach in defining innovativeness of a new medicine.

		**Added therapeutic value**				
	***n***	**Maximum**	**Important**	**Moderate**	**Poor**	**Absent**
Therapeutic need	Maximum	1	5	4	3	0
	Important	0	12	14	6	2
	Moderate	0	15	20	16	1
	Poor	0	0	1	4	0
	Absent	0	0	0	0	0
		**Therapeutic need**				
	***n***	**Maximum**	**Important**	**Moderate**	**Poor**	**Absent**
Quality of clinical evidence	High	1	8	9	0	0
	Moderate	4	15	38	4	0
	Low	6	11	5	0	0
	Very low	2	2	3	1	0
		**Added therapeutic value**				
	***n***	**Maximum**	**Important**	**Moderate**	**Poor**	**Absent**
Quality of clinical evidence	High	0	6	7	3	2
	Moderate	1	17	19	20	1
	Low	0	8	8	5	0
	Very low	0	1	5	1	0

*Cramer V = 0.24; p = 0.224. Cramer V = 0.25; p = 0.008. Cramer V = 0.20; p = 0.517. Data were summarized as numbers (n). Chi-squared test was used to compute the p-value for Cramer's V*.

The analysis of all possible combinations of the three criteria used to define drug innovativeness showed 37 different evaluation profiles; the most frequent combination (13.8%) was represented by the “moderate” level reached for all three criteria, followed by the combination Moderate/Poor/Moderate and Moderate/Important/Moderate with the same percentage (9.0%) (Table 3). Furthermore, in most cases, the same profile observed was linked to the same final decision. For example, the 15 therapeutic indications rated as “Moderate/Moderate/Moderate” were classified always as conditionally innovative while the other two most frequent profiles “Moderate/Poor/Moderate” and “Moderate/Important/Moderate” were classified always as non-innovative and fully innovative, respectively. From these combinations, it was possible to observe also the role played by the quality of clinical evidence criterion. In fact, considering the two different patterns “Important/Moderate/High” (*n* = 5; 4.6%) and “Important/Moderate/Low” (*n* = 4; 3.7%) which differ only for the quality level of clinical evidence, the majority of reports were classified as fully innovative (80%) in the first case, whereas in the second case they were classified either as conditionally innovative (50%) or as non-innovative (50%). Furthermore, the observed criteria combination patterns were not so much altered when separating orphans from non-orphan drugs (Supplementary Table 3) and oncological from non-oncological drugs (Supplementary Table 6).

**TABLE 3:** Criteria combination patterns in relation to drug innovativeness definition.

**Therapeutic need**	**Added therapeutic value**	**Quality of clinical evidence**	***n***	**Fully innovative (%)**	**Conditionally innovative (%)**	**Non-innovative (%)**
Moderate	Moderate	Moderate	15	0	100	0
Moderate	Poor	Moderate	10	0	0	100
Moderate	Important	Moderate	10	100	0	0
Important	Important	Moderate	6	100	0	0
Important	Moderate	High	5	80	20	0
Poor	Poor	Moderate	4	0	0	100
Moderate	Important	High	4	100	0	0
Important	Poor	Moderate	4	0	0	100
Important	Moderate	Low	4	0	50	50
Important	Moderate	Moderate	4	25	75	0
Important	Important	Low	4	100	0	0
Moderate	Poor	High	3	0	0	100
Moderate	NA	Moderate	3	0	0	100
Maximum	Important	Low	3	67	0	33
Moderate	Poor	Low	2	0	0	100
Moderate	Moderate	Very low	2	0	0	100
Moderate	Moderate	Low	2	0	100	0
Important	Poor	Low	2	0	0	100
Important	Important	High	2	100	0	0
Maximum	Poor	Moderate	2	0	0	100
Maximum	Moderate	Low	2	0	100	0
Poor	Moderate	Very low	1	0	0	100
Moderate	Absent	High	1	0	0	100
Moderate	Poor	Very low	1	0	0	100
Moderate	Moderate	High	1	0	100	0
Moderate	Important	Low	1	100	0	0
Important	Absent	Moderate	1	0	0	100
Important	Absent	High	1	0	0	100
Important	Moderate	Very low	1	0	100	0
Important	NA	Very low	1	0	0	100
Important	NA	Low	1	0	0	100
Maximum	Poor	Low	1	0	0	100
Maximum	Moderate	Very low	1	0	100	0
Maximum	Moderate	High	1	0	100	0
Maximum	Important	Very low	1	100	0	0
Maximum	Important	Moderate	1	100	0	0
Maximum	Maximum	Moderate	1	100	0	0
			109			

*Combination patterns are ordered by decreasing frequency (n)*.

**Figure 1:**
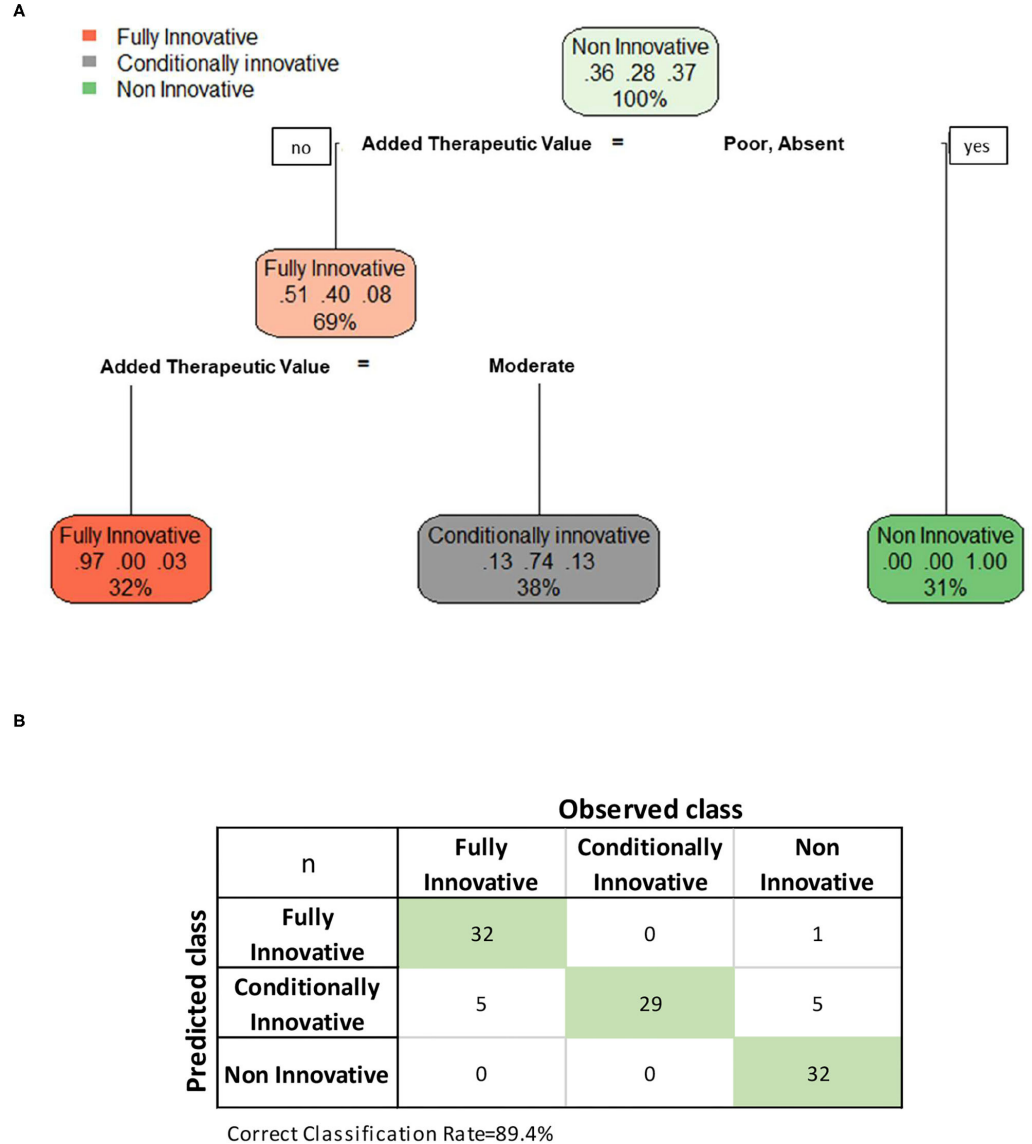
**(A)** Illustration of classification tree built according to recursive partitioning (RPART)^3^ model for the evaluation of drug's innovation and **(B)** confusion matrix of the predicted vs observed classification responses for the complete sample without missing values (*n* = 104). In each node are reported three main pieces of information: in the first line the name of the “most” frequent category of the outcome variable, in the second line the percentage for each category on the total amount of node observations, and in the third line the percentage of observations within the node on the total amount used in the model (*n* = 104). For example, in the root node, the most frequent outcome's category is “Non-innovative,” with a percentage of 37%. The root node contains the total amount of observations (100%) used in the model. While in the splitting node the most frequent outcome's category is “Fully innovative” for 51% of nodes observations which contains 69% of the total amount of observations.

The CT model confirmed the previous results. As shown by Figure 1, the added therapeutic value was the most important variable in predicting the innovativeness status. In the first split, the “poor” or “absent” level of the added therapeutic value distinguished a final node of 32 reports whose predicted class was no innovativeness. In the second split, the remaining 72 reports were divided into two final nodes according to the following rule: “maximum” or “important” added therapeutic values distinguished a final node of 33 reports whose predicted class was fully innovative, otherwise the predicted class was conditional innovativeness. The CT achieved an accuracy of 89.4% for the entire sample. Unaltered results were observed repeating the CT model and splitting the entire sample in training and test samples (results not shown).

## Discussion

To date, this study represents the most updated analysis of the AIFA's assessment reports of drug innovativeness, since the new approach using GRADE methodology was adopted by AIFA in order to evaluate the innovativeness of medications in Italy.

First of all, our study showed that the type of medicine, oncological or orphan drug, does not influence the final decision on drug innovativeness, suggesting that these characteristics cannot be considered as determinants of drug innovativeness.

We know that drugs may be considered “Fully Innovative” if both the therapeutic need and the added therapeutic value were recognized as “Maximum” or “Important,” and the quality of the clinical evidence as “High.” On the other hand, a drug cannot be recognized innovative if the therapeutic need and/or the added therapeutic value was judged as “poor” or “absent,” or if the quality of clinical evidence was judged to be “low” or “very low” (with the exception of the orphan drugs, which can achieve the “Fully Innovative” status even with low or very low quality of clinical evidence).

Intermediate situations are evaluated case by case, taking into account the relative weight of each criterion. We found that the added therapeutic value was the only criteria statistically associated with drug innovation degree, suggesting that it is the most important criterion for the definition of the innovative status of a new medicine or a new therapeutic indication. This means that the rate of the clinical benefits is the determinant of the innovativeness status and that the extent of clinical efficacy of a medicine, measured on clinically relevant endpoints, when compared to the available alternative treatments, is the crucial factor in recognizing a medicine as fully innovative. This association was confirmed also when separating orphan drugs from non-orphans and oncological drugs from non-oncological ones.

Moreover, we found that if the added therapeutic value is moderate, then the quality of clinical evidence has a determinant role in the decision. The therapeutic need, evaluated as the availability of alternative therapies, seems to have no determinant role in the final decision.

The analysis of all possible combinations of the three criteria used to define innovativeness of a drug showed a mild association between two criteria of the AIFA's approach, the therapeutic need, and the quality of clinical evidence. In most cases the same observed profile was linked to the same decision on drug innovativeness, thus we can assume that similar decision profiles bring to the same evaluation of innovativeness status, indicating a good consistency between decisions. This applies also to analyzing orphans/non-orphans and oncological/non-oncological drugs separately.

Based on the publicly available assessment reports, a classification tree model was used to understand how the framework works as well as to identify the most important criteria in predicting the final decision. The CT model confirmed that the added therapeutic value was the most important determinant on innovativeness decision, followed by quality of evidence, mainly when the added therapeutic value was evaluated as moderate. In addition, the therapeutic need seems to have no relevant role in the evaluation. Unaltered results were observed when repeating the CT model by splitting the entire sample in training and test samples, thus showing good performance of the model in predicting the final decision.

Our study confirms the results of a previously published paper (16), including a limited number of appraisals, and showing that the added therapeutic value has the greatest impact on the final decision of the appraisal of drug innovativeness. However, our analysis was more extensive than the previous one, because it was based on a larger number of appraisals and included the evaluation of the potential interdependence between criteria and the application of a classification tree model, which was found to efficiently predict the final decision.

As far as the decisions on drug innovativeness can be arbitrary, this study confirmed that the AIFA's multidimensional approach, which is well-structured and flexible at the same time, provides a systematic approach in the assessment in order to minimize biases and improve consistency of the decisions. The application of the three criteria in a decision framework, including the GRADE methodology in evaluating the quality of clinical evidence, goes in the direction of improving the transparency and reproducibility of the decision-making process on drug innovativeness.

Beyond a few methodological and procedural differences, the Italian model for drug innovativeness designation (3) could be considered quite similar to systems used in other European countries, in particular in the evaluation of the added therapeutic value. For example, in France and Italy, the added therapeutic value can range across 5 levels, whereas in Germany six categories were identified (17, 18). However, in Italy, the added therapeutic value of a medicine represents the leading criterion for the definition of drug innovativeness, whereas in France and Germany the evaluation of the added health-related benefits poses the basis for the negotiation of the reimbursed price/discounts (19, 20). In addition, some similarities exist between these rankings and the way to measure the added therapeutic value of a medicine in the NICE approach for appraising innovative technologies in the UK (21, 22).

Furthermore, in Italy, the quality of evidence is assessed through the GRADE approach and ranked in four levels. In Germany, both the number and characteristics of clinical studies as well as the certainty of results and observed effects are ranked in four classes of evidence quality (23). In France, the quality of studies is taken into account, although without a defined classification system. In Italy and France, surrogate endpoints are accepted if no other endpoints are available; in Germany, these are considered only if validated. Finally, Italy and Germany apply specific rules (e.g., accepting a low level of quality of evidence) for orphan medicines (24, 25).

Even in the USA, the breakthrough therapy designation by Food and Drug Administration (FDA) was created in 2012 to expedite the development and review of drugs intended to treat a serious or life-threatening disease that, based on preliminary evidence, were expected to provide substantial benefits over existing therapies on a clinically significant endpoint(s) (26, 27).

Although with a different role played by the three criteria within the HTA process in the various countries, there is some evidence of a harmonized approach to recognize an innovative medicine, which should guarantee equity in the access to innovative medicines for European patients. Moreover, we would highlight that, within the context of the European countries, Italy is the only one where the “innovative status” is granted on a well-structured and reproducible model, with a list of innovative (and non-innovative) medicines made publicly available.

Despite the fact that our study results should be consolidated through further analyses based on a larger sample of appraisals, our study showed the benefits of the model adopted in Italy for drug “innovative” designation for decision-makers (AIFA), who would have an efficient and transparent methodology to make decisions on drug innovativeness and for patients, who could have more rapid access to new innovative medicines.

In conclusion, this article provides an extensive overview of AIFA's published assessment reports on drug innovativeness in Italy, identifying the determinants of drug innovativeness and both the accuracy and consistency of the assessments across the last 3 years. We confirmed that the multidimensional approach chosen by AIFA since April 2017 reached the intended purpose of the Italian regulatory Agency both in terms of transparency and accountability of the decision-making process applied to innovative medicines.

## Data Availability Statement

The datasets presented in this study can be found in online repositories. The names of the repository/repositories and accession number(s) can be found below: https://www.aifa.gov.it/web/guest/farmaci-innovativi.

## Author Contributions

SP, FF, and FT: conception and study design. FF: collection and assembly of data. SP, RM, and FF: analysis and interpretation. FT, MT, and AD: methodological advice. FF and SP: manuscript writing. All authors read, revised, and approved the final manuscript.

## Conflict of Interest

The authors declare that the research was conducted in the absence of any commercial or financial relationships that could be construed as a potential conflict of interest.

## Publisher's Note

All claims expressed in this article are solely those of the authors and do not necessarily represent those of their affiliated organizations, or those of the publisher, the editors and the reviewers. Any product that may be evaluated in this article, or claim that may be made by its manufacturer, is not guaranteed or endorsed by the publisher.
