# Supplementary material for: The Assessment of the Innovativeness of a New Medicine in Italy
Source: Front Med (Lausanne). 2021 Dec 8;8:793640. doi: 10.3389/fmed.2021.793640 (PMC8692651; doi:10.3389/fmed.2021.793640)
Supplement: Supplementary file 1 [file Data_Sheet_1.docx]

# Supplementary files - The assessment of the innovativeness of a new medicine product in Italy

**Table 1** -Characteristics of orphan and non-orphan drugs considering the drug’s degree of innovation.

|  |  | **Orphan drugs (n=41)** | | | | | | | | |  | **Non-Orphan drugs (n=68)** | | | | | | | | |
| --- | --- | --- | --- | --- | --- | --- | --- | --- | --- | --- | --- | --- | --- | --- | --- | --- | --- | --- | --- | --- |
|  |  | **Fully Innovative** | |  | **Conditionally Innovative** | |  | **Non Innovative** | | **p-value*** |  | **Fully Innovative** | |  | **Conditionally Innovative** | |  | **Non Innovative†** | | **p-value*** |
|  |  | n=16 | |  | n=11 | |  | n=14 | |  |  | n=21 | |  | n=18 | |  | n=29 | |  |
| **Oncological drugs** | | 10 | 62,5 |  | 6 | 54,5 |  | 8 | 57,1 | 0,925 |  | 14 | 66,7 |  | 14 | 77,8 |  | 15 | 51,7 | 0,193 |
| **Therapeutic Need** | |  |  |  |  |  |  |  |  |  |  |  |  |  |  |  |  |  |  |  |
|  | Maximum | 4 | 25,0 |  | 1 | 9,1 |  | 3 | 21,4 | 0,327 |  | 1 | 4,8 |  | 3 | 16,7 |  | 1 | 3,4 | 0,200 |
|  | Important | 6 | 37,5 |  | 3 | 27,3 |  | 3 | 21,4 |  |  | 11 | 52,4 |  | 4 | 22,2 |  | 9 | 31,0 |  |
|  | Moderate | 6 | 37,5 |  | 7 | 63,6 |  | 5 | 35,7 |  |  | 9 | 42,9 |  | 11 | 61,1 |  | 17 | 58,6 |  |
|  | Poor | 0 | 0,0 |  | 0 | 0,0 |  | 3 | 21,4 |  |  | 0 | 0,0 |  | 0 | 0,0 |  | 2 | 6,9 |  |
|  | Absent | 0 | 0,0 |  | 0 | 0,0 |  | 0 | 0,0 |  |  | 0 | 0,0 |  | 0 | 0,0 |  | 0 | 0,0 |  |
| **Added Therapeutic Value** | |  |  |  |  |  |  |  | 0,0 |  |  |  |  |  |  |  |  |  |  |  |
|  | Maximum | 0 | 0,0 |  | 0 | 0,0 |  | 0 | 0,0 | <0.001 |  | 1 | 4,8 |  | 0 | 0,0 |  | 0 | 0,0 | <0.001 |
|  | Important | 16 | 100,0 |  | 0 | 0,0 |  | 1 | 7,1 |  |  | 15 | 71,4 |  | 0 | 0,0 |  | 0 | 0,0 |  |
|  | Moderate | 0 | 0,0 |  | 11 | 100,0 |  | 4 | 28,6 |  |  | 5 | 23,8 |  | 18 | 100,0 |  | 1 | 4,2 |  |
|  | Poor | 0 | 0,0 |  | 0 | 0,0 |  | 9 | 64,3 |  |  | 0 | 0,0 |  | 0 | 0,0 |  | 20 | 83,3 |  |
|  | Absent | 0 | 0,0 |  | 0 | 0,0 |  | 0 | 0,0 |  |  | 0 | 0,0 |  | 0 | 0,0 |  | 3 | 12,5 |  |
| **Quality of Clinical Evidence** | |  |  |  |  |  |  |  | 0,0 |  |  |  |  |  |  |  |  |  |  |  |
|  | High | 1 | 6,3 |  | 1 | 9,1 |  | 0 | 0,0 | 0,715 |  | 9 | 42,9 |  | 2 | 11,1 |  | 5 | 17,2 | 0,265 |
|  | Moderate | 9 | 56,3 |  | 6 | 54,5 |  | 5 | 35,7 |  |  | 10 | 47,6 |  | 12 | 66,7 |  | 19 | 65,5 |  |
|  | Low | 5 | 31,3 |  | 3 | 27,3 |  | 6 | 42,9 |  |  | 2 | 9,5 |  | 3 | 16,7 |  | 3 | 10,3 |  |
|  | Very low | 1 | 6,3 |  | 1 | 9,1 |  | 3 | 21,4 |  |  | 0 | 0,0 |  | 1 | 5,6 |  | 2 | 6,9 |  |
| Data were summarized as numbers (n) and frequencies (%). | | | | | | | | | | | | | | | | | | | | |
| *Fisher’s exact test was applied to evaluate the association between categorical variables. | | | | | | | | | | | | | | | | | | | | |
| † For one observation the Added Therapeutic Value was "Untestable" and therefore classified as NA. | | | | | | | | | | | | | | | | | | | | |

**Table 2** - Relationship between criteria utilized in the multidimensional approach in defining innovativeness of a new medicine product considering (a) orphan and (b) non-orphan drugs

| **(a) Orphan drugs (n=41)** | | |  |  |  |  |  | **(b) Non-orphan drugs (n=68)** | | |  |  |  |  |
| --- | --- | --- | --- | --- | --- | --- | --- | --- | --- | --- | --- | --- | --- | --- |
|  |  | **Added Therapeutic Value** | | | | |  |  |  | **Added Therapeutic Value** | | | | |
| **Therapeutic Need** | n | Maximum | Important | Moderate | Poor | Absent |  | **Therapeutic Need** | N | Maximum | Important | Moderate | Poor | Absent |
|  | Maximum | 0 | 5 | 1 | 2 | 0 |  |  | Maximum | 1 | 0 | 3 | 1 | 0 |
|  | Important | 0 | 6 | 4 | 2 | 0 |  |  | Important | 0 | 6 | 10 | 4 | 2 |
|  | Moderate | 0 | 6 | 9 | 3 | 0 |  |  | Moderate | 0 | 9 | 11 | 13 | 1 |
|  | Poor | 0 | 0 | 1 | 2 | 0 |  |  | Poor | 0 | 0 | 0 | 2 | 0 |
|  | Absent | 0 | 0 | 0 | 0 | 0 |  |  | Absent | 0 | 0 | 0 | 0 | 0 |
| Cramer V=0.31; p-value=0.295 | | |  |  |  |  |  | Cramer V=0.34; p-value=0.152 | | |  |  |  |  |
|  |  |  |  |  |  |  |  |  |  |  |  |  |  |  |
|  |  | **Therapeutic Need** | | | | |  |  |  | **Therapeutic Need** | | | | |
| **Quality of Clinical Evidence** | n | Maximum | Important | Moderate | Poor | Absent |  | **Quality of Clinical Evidence** | N | Maximum | Important | Moderate | Poor | Absent |
|  | High | 0 | 0 | 2 | 0 | 0 |  |  | High | 1 | 8 | 7 | 0 | 0 |
|  | Moderate | 2 | 5 | 11 | 2 | 0 |  |  | Moderate | 2 | 10 | 27 | 2 | 0 |
|  | Low | 4 | 7 | 3 | 0 | 0 |  |  | Low | 2 | 4 | 2 | 0 | 0 |
|  | Very Low | 2 | 0 | 2 | 1 | 0 |  |  | Very Low | 0 | 2 | 1 | 0 | 0 |
| Cramer V=0.33; p-value=0.096 | | |  |  |  |  |  | Cramer V=0.24; p-value=0.171 | | |  |  |  |  |
|  |  |  |  |  |  |  |  |  |  |  |  |  |  |  |
|  |  | **Added Therapeutic Value** | | | | |  |  |  | **Added Therapeutic Value** | | | | |
| **Quality of Clinical Evidence** | n | Maximum | Important | Moderate | Poor | Absent |  | **Quality of Clinical Evidence** | N | Maximum | Important | Moderate | Poor | Absent |
|  | High | 0 | 1 | 1 | 0 | 0 |  |  | High | 0 | 5 | 6 | 3 | 2 |
|  | Moderate | 0 | 9 | 6 | 5 | 0 |  |  | Moderate | 1 | 8 | 13 | 15 | 1 |
|  | Low | 0 | 6 | 4 | 4 | 0 |  |  | Low | 0 | 2 | 4 | 1 | 0 |
|  | Very Low | 0 | 1 | 4 | 0 | 0 |  |  | Very Low | 0 | 0 | 1 | 1 | 0 |
| Cramer V=0.26; p-value=0.574 | | |  |  |  |  |  | Cramer V=0.20; p-value=0.693 | | |  |  |  |  |
|  |  |  |  |  |  |  |  |  |  |  |  |  |  |  |
| Data were summarized as numbers (n). | | | |  |  |  |  |  |  |  |  |  |  |  |
| Chi squared test was used to compute the p-value for Cramer's V. | | | | | | |  |  |  |  |  |  |  |  |
| † For five observation the Added Therapeutic Value was "Untestable" and therefore classified as NA. | | | | | | | | | |  |  |  |  |  |

| **Table 3**-Criteria combination patterns in relation with drug innovativeness definition by orphan and non-orphan drugs. Combination patterns are ordered by decreasing frequency (n). | | | | | | | |
| --- | --- | --- | --- | --- | --- | --- | --- |
| **Therapeutic Need** | **Added Therapeutic Value** | **Quality of Clinical Evidence** | **N** |  | **Fully Innovative (%)** | **Conditionally Innovative (%)** | **Non Innovative (%)** |
| **• Orphan drugs** | |  |  |  |  |  |  |
| Moderate | Moderate | Moderate | 5 |  | 0 | 100 | 0 |
| Moderate | Important | Moderate | 5 |  | 100 | 0 | 0 |
| Important | Moderate | Low | 3 |  | 0 | 67 | 33 |
| Important | Important | Low | 3 |  | 100 | 0 | 0 |
| Important | Important | Moderate | 3 |  | 100 | 0 | 0 |
| Maximum | Important | Low | 3 |  | 67 | 0 | 33 |
| Poor | Poor | Moderate | 2 |  | 0 | 0 | 100 |
| Moderate | Poor | Low | 2 |  | 0 | 0 | 100 |
| Moderate | Moderate | Very low | 2 |  | 0 | 0 | 100 |
| Poor | Moderate | Very low | 1 |  | 0 | 0 | 100 |
| Moderate | Poor | Moderate | 1 |  | 0 | 0 | 100 |
| Moderate | Moderate | Low | 1 |  | 0 | 100 | 0 |
| Moderate | Moderate | High | 1 |  | 0 | 100 | 0 |
| Moderate | Important | High | 1 |  | 100 | 0 | 0 |
| Important | Poor | Low | 1 |  | 0 | 0 | 100 |
| Important | Poor | Moderate | 1 |  | 0 | 0 | 100 |
| Important | Moderate | Moderate | 1 |  | 0 | 100 | 0 |
| Maximum | Poor | Low | 1 |  | 0 | 0 | 100 |
| Maximum | Poor | Moderate | 1 |  | 0 | 0 | 100 |
| Maximum | Moderate | Very low | 1 |  | 0 | 100 | 0 |
| Maximum | Important | Very low | 1 |  | 100 | 0 | 0 |
| Maximum | Important | Moderate | 1 |  | 100 | 0 | 0 |
| **• Non-orphan drugs** | |  | **41** |  |  |  |  |
| Moderate | Moderate | Moderate | 10 |  | 0 | 100 | 0 |
| Moderate | Poor | Moderate | 9 |  | 0 | 0 | 100 |
| Moderate | Important | Moderate | 5 |  | 100 | 0 | 0 |
| Important | Moderate | High | 5 |  | 80 | 20 | 0 |
| Moderate | Poor | High | 3 |  | 0 | 0 | 100 |
| Moderate | Important | High | 3 |  | 100 | 0 | 0 |
| Moderate | NA | Moderate | 3 |  | 0 | 0 | 100 |
| Important | Poor | Moderate | 3 |  | 0 | 0 | 100 |
| Important | Moderate | Moderate | 3 |  | 33 | 67 | 0 |
| Important | Important | Moderate | 3 |  | 100 | 0 | 0 |
| Poor | Poor | Moderate | 2 |  | 0 | 0 | 100 |
| Important | Important | High | 2 |  | 100 | 0 | 0 |
| Maximum | Moderate | Low | 2 |  | 0 | 100 | 0 |
| Moderate | Absent | High | 1 |  | 0 | 0 | 100 |
| Moderate | Poor | Very low | 1 |  | 0 | 0 | 100 |
| Moderate | Moderate | Low | 1 |  | 0 | 100 | 0 |
| Moderate | Important | Low | 1 |  | 100 | 0 | 0 |
| Important | Absent | Moderate | 1 |  | 0 | 0 | 100 |
| Important | Absent | High | 1 |  | 0 | 0 | 100 |
| Important | Poor | Low | 1 |  | 0 | 0 | 100 |
| Important | Moderate | Very low | 1 |  | 0 | 100 | 0 |
| Important | Moderate | Low | 1 |  | 0 | 0 | 100 |
| Important | Important | Low | 1 |  | 100 | 0 | 0 |
| Important | NA | Very low | 1 |  | 0 | 0 | 100 |
| Important | NA | Low | 1 |  | 0 | 0 | 100 |
| Maximum | Poor | Moderate | 1 |  | 0 | 0 | 100 |
| Maximum | Moderate | High | 1 |  | 0 | 100 | 0 |
| Maximum | Maximum | Moderate | 1 |  | 100 | 0 | 0 |
|  |  |  | **68** |  |  |  |  |

**Table 4** - Characteristics of oncological and non-oncological drugs considering the drug’s degree of innovation

|  |  | **Oncological drugs (n=67)** | | | | | | | | |  | **Non-Oncological drugs (n=42)** | | | | | | | | |
| --- | --- | --- | --- | --- | --- | --- | --- | --- | --- | --- | --- | --- | --- | --- | --- | --- | --- | --- | --- | --- |
|  |  | **Fully Innovative** | |  | **Conditionally Innovative** | |  | **Non Innovative†** | | **p-value*** |  | **Fully Innovative** | |  | **Conditionally Innovative** | |  | **Non Innovative** | | **p-value*** |
|  |  | n=24 | |  | n=20 | |  | n=23 | |  |  | n=13 | |  | n=9 | |  | n=20 | |  |
| **Orphan drugs** | | 10 | 62,5 |  | 6 | 54,5 |  | 8 | 57,1 | 0,770 |  | 6 | 46,2 |  | 6 | 66,7 |  | 6 | 30,0 | 0,396 |
| **Therapeutic Need** | |  |  |  |  |  |  |  |  |  |  |  |  |  |  |  |  |  |  |  |
|  | Maximum | 1 | 4,2 |  | 3 | 15,0 |  | 1 | 4,3 | 0,284 |  | 4 | 30,8 |  | 1 | 11,1 |  | 3 | 15,0 | 0,058 |
|  | Important | 10 | 41,7 |  | 4 | 16,7 |  | 9 | 39,1 |  |  | 7 | 53,8 |  | 3 | 33,3 |  | 3 | 15,0 |  |
|  | Moderate | 13 | 54,2 |  | 13 | 54,2 |  | 11 | 47,8 |  |  | 2 | 15,4 |  | 5 | 55,6 |  | 11 | 55,0 |  |
|  | Poor | 0 | 0,0 |  | 0 | 0,0 |  | 2 | 8,7 |  |  | 0 | 0,0 |  | 0 | 0,0 |  | 3 | 15,0 |  |
|  | Absent | 0 | 0,0 |  | 0 | 0,0 |  | 0 | 0,0 |  |  | 0 | 0,0 |  | 0 | 0,0 |  | 0 | 0,0 |  |
| **Added Therapeutic Value** | |  |  |  |  |  |  |  |  |  |  |  |  |  |  |  |  |  |  |  |
|  | Maximum | 0 | 0,0 |  | 0 | 0,0 |  | 0 | 0,0 | <0.001 |  | 1 | 7,7 |  | 0 | 0,0 |  | 0 | 0,0 | <0.001 |
|  | Important | 21 | 87,5 |  | 0 | 0,0 |  | 1 | 5,6 |  |  | 10 | 76,9 |  | 0 | 0,0 |  | 0 | 0,0 |  |
|  | Moderate | 3 | 12,5 |  | 20 | 100,0 |  | 3 | 16,7 |  |  | 2 | 15,4 |  | 9 | 100,0 |  | 2 | 10,0 |  |
|  | Poor | 0 | 0,0 |  | 0 | 0,0 |  | 12 | 66,7 |  |  | 0 | 0,0 |  | 0 | 0,0 |  | 17 | 85,0 |  |
|  | Absent | 0 | 0,0 |  | 0 | 0,0 |  | 2 | 11,1 |  |  | 0 | 0,0 |  | 0 | 0,0 |  | 1 | 5,0 |  |
| **Quality of Clinical Evidence** | |  |  |  |  |  |  |  |  |  |  |  |  |  |  |  |  |  |  |  |
|  | High | 8 | 33,3 |  | 3 | 15,0 |  | 1 | 4,3 | 0,057 |  | 2 | 15,4 |  | 0 | 0,0 |  | 4 | 20,0 | 0,548 |
|  | Moderate | 14 | 58,3 |  | 14 | 70,0 |  | 13 | 56,5 |  |  | 5 | 38,5 |  | 4 | 44,4 |  | 11 | 55,0 |  |
|  | Low | 2 | 8,3 |  | 2 | 10,0 |  | 6 | 26,1 |  |  | 5 | 38,5 |  | 4 | 44,4 |  | 3 | 15,0 |  |
|  | Very low | 0 | 0,0 |  | 1 | 5,0 |  | 3 | 13,0 |  |  | 1 | 7,7 |  | 1 | 11,1 |  | 2 | 10,0 |  |
| Data were summarized as numbers (n) and frequencies (%). | | | | | | | | | | | | | | | | | | | | |
| *Fisher’s exact test was applied to evaluate the association between categorical variables. | | | | | | | | | | | | | | | | | | | | |
| † For one observation the Added Therapeutic Value was "Untestable" and therefore classified as NA. | | | | | | | | | | | | | | | | | | | | |

**Table 5** - Relationship between criteria utilized in the multidimensional approach in defining innovativeness of a new medicine product considering (a) oncological and (b) non-oncological drugs

| **(a) Oncological drugs† (n=67)** | | | |  |  |  |  | **(b) Non-oncological drugs (n=42)** | | | |  |  |  |
| --- | --- | --- | --- | --- | --- | --- | --- | --- | --- | --- | --- | --- | --- | --- |
|  |  | **Added Therapeutic Value** | | | | |  |  |  | **Added Therapeutic Value** | | | | |
| **Therapeutic Need** | n | Maximum | Important | Moderate | Poor | Absent |  | **Therapeutic Need** | n | Maximum | Important | Moderate | Poor | Absent |
|  | Maximum | 0 | 2 | 3 | 0 | 0 |  |  | Maximum | 1 | 3 | 1 | 3 | 0 |
|  | Important | 0 | 7 | 8 | 4 | 2 |  |  | Important | 0 | 5 | 6 | 2 | 0 |
|  | Moderate | 0 | 13 | 15 | 6 | 0 |  |  | Moderate | 0 | 2 | 5 | 10 | 1 |
|  | Poor | 0 | 0 | 0 | 2 | 0 |  |  | Poor | 0 | 0 | 1 | 2 | 0 |
|  | Absent | 0 | 0 | 0 | 0 | 0 |  |  | Absent | 0 | 0 | 0 | 0 | 0 |
| Cramer V = 0.27; p-value=0.243 | | |  |  |  |  |  | Cramer V = 0.34; p-value=0.138 | | |  |  |  |  |
|  |  |  |  |  |  |  |  |  |  |  |  |  |  |  |
|  |  | **Therapeutic Need** | | | | |  |  |  | **Therapeutic Need** | | | | |
| **Quality of Clinical Evidence** | n | Maximum | Important | Moderate | Poor | Absent |  | **Quality of Clinical Evidence** | n | Maximum | Important | Moderate | Poor | Absent |
|  | High | 1 | 7 | 4 | 0 | 0 |  |  | High | 0 | 1 | 5 | 0 | 0 |
|  | Moderate | 1 | 8 | 30 | 2 | 0 |  |  | Moderate | 3 | 7 | 8 | 2 | 0 |
|  | Low | 3 | 6 | 1 | 0 | 0 |  |  | Low | 3 | 5 | 4 | 0 | 0 |
|  | Very Low | 0 | 2 | 2 | 0 | 0 |  |  | Very Low | 2 | 0 | 1 | 1 | 0 |
| Cramer V =0.34; p-value=0.003 | | |  |  |  |  |  | Cramer V =0.31; p-value=0.272 | | |  |  |  |  |
|  |  |  |  |  |  |  |  |  |  |  |  |  |  |  |
|  |  | **Added Therapeutic Value** | | | | |  |  |  | **Added Therapeutic Value** | | | | |
| **Quality of Clinical Evidence** | n | Maximum | Important | Moderate | Poor | Absent |  | **Quality of Clinical Evidence** | n | Maximum | Important | Moderate | Poor | Absent |
|  | High | 0 | 5 | 6 | 0 | 1 |  |  | High | 0 | 1 | 1 | 3 | 1 |
|  | Moderate | 0 | 14 | 14 | 9 | 1 |  |  | Moderate | 1 | 3 | 5 | 11 | 0 |
|  | Low | 0 | 3 | 3 | 3 | 0 |  |  | Low | 0 | 5 | 5 | 2 | 0 |
|  | Very Low | 0 | 0 | 3 | 0 | 0 |  |  | Very Low | 0 | 1 | 2 | 1 | 0 |
| Cramer V =0.23; p-value=0.339 | | |  |  |  |  |  | Cramer V =0.34; p-value=0.297 | | |  |  |  |  |
|  |  |  |  |  |  |  |  |  |  |  |  |  |  |  |
| Data were summarized as numbers (n). | | | |  |  |  |  |  |  |  |  |  |  |  |
| Chi squared test was used to compute the p-value for Cramer's V. | | | | | | |  |  |  |  |  |  |  |  |
| † For five observation the Added Therapeutic Value was "Untestable" and therefore classified as NA. | | | | | | | | | |  |  |  |  |  |

**Table 6** - Criteria combination patterns in relation with drug innovativeness definition by oncological and non-oncological drugs. Combination patterns are ordered by decreasing frequency (n).

| **Therapeutic Need** | **Added Therapeutic Value** | **Quality of Clinical Evidence** | **n** |  | **Fully Innovative (%)** | **Conditionally Innovative (%)** | **Non Innovative (%)** |
| --- | --- | --- | --- | --- | --- | --- | --- |
| **• Oncological drugs** | |  |  |  |  |  |  |
| Moderate | Moderate | Moderate | 12 |  | 0 | 100 | 0 |
| Moderate | Important | Moderate | 10 |  | 100 | 0 | 0 |
| Moderate | Poor | Moderate | 5 |  | 0 | 0 | 100 |
| Important | Moderate | High | 4 |  | 75 | 25 | 0 |
| Moderate | Important | High | 3 |  | 100 | 0 | 0 |
| Moderate | NA | Moderate | 3 |  | 0 | 0 | 100 |
| Important | Important | Moderate | 3 |  | 100 | 0 | 0 |
| Poor | Poor | Moderate | 2 |  | 0 | 0 | 100 |
| Moderate | Moderate | Very Low | 2 |  | 0 | 0 | 100 |
| Important | Poor | Low | 2 |  | 0 | 0 | 100 |
| Important | Poor | Moderate | 2 |  | 0 | 0 | 100 |
| Important | Moderate | Moderate | 2 |  | 0 | 100 | 0 |
| Important | Important | Low | 2 |  | 100 | 0 | 0 |
| Important | Important | High | 2 |  | 100 | 0 | 0 |
| Maximum | Moderate | Low | 2 |  | 0 | 100 | 0 |
| Moderate | Poor | Low | 1 |  | 0 | 0 | 100 |
| Moderate | Moderate | High | 1 |  | 0 | 100 | 0 |
| Important | Absent | Moderate | 1 |  | 0 | 0 | 100 |
| Important | Absent | High | 1 |  | 0 | 0 | 100 |
| Important | Moderate | Very Low | 1 |  | 0 | 100 | 0 |
| Important | Moderate | Low | 1 |  | 0 | 0 | 100 |
| Important | NA | Very Low | 1 |  | 0 | 0 | 100 |
| Important | NA | Low | 1 |  | 0 | 0 | 100 |
| Maximum | Moderate | High | 1 |  | 0 | 100 | 0 |
| Maximum | Important | Low | 1 |  | 0 | 0 | 100 |
| Maximum | Important | Moderate | 1 |  | 100 | 0 | 0 |
| **• Non - oncological drugs** | | | **67** |  |  |  |  |
| Moderate | Poor | Moderate | 5 |  | 0 | 0 | 100 |
| Moderate | Poor | High | 3 |  | 0 | 0 | 100 |
| Moderate | Moderate | Moderate | 3 |  | 0 | 100 | 0 |
| Important | Moderate | Low | 3 |  | 0 | 67 | 33 |
| Important | Important | Moderate | 3 |  | 100 | 0 | 0 |
| Poor | Poor | Moderate | 2 |  | 0 | 0 | 100 |
| Moderate | Moderate | Low | 2 |  | 0 | 100 | 0 |
| Important | Poor | Moderate | 2 |  | 0 | 0 | 100 |
| Important | Moderate | Moderate | 2 |  | 50 | 50 | 0 |
| Important | Important | Low | 2 |  | 100 | 0 | 0 |
| Maximum | Poor | Moderate | 2 |  | 0 | 0 | 100 |
| Maximum | Important | Low | 2 |  | 100 | 0 | 0 |
| Poor | Moderate | Very Low | 1 |  | 0 | 0 | 100 |
| Moderate | Absent | High | 1 |  | 0 | 0 | 100 |
| Moderate | Poor | Very Low | 1 |  | 0 | 0 | 100 |
| Moderate | Poor | Low | 1 |  | 0 | 0 | 100 |
| Moderate | Important | Low | 1 |  | 100 | 0 | 0 |
| Moderate | Important | High | 1 |  | 100 | 0 | 0 |
| Important | Moderate | High | 1 |  | 100 | 0 | 0 |
| Maximum | Poor | Low | 1 |  | 0 | 0 | 100 |
| Maximum | Moderate | Very Low | 1 |  | 0 | 100 | 0 |
| Maximum | Important | Very Low | 1 |  | 100 | 0 | 0 |
| Maximum | Maximum | Moderate | 1 |  | 100 | 0 | 0 |
|  |  |  | **42** |  |  |  |  |
